# Patient-centered consultations for persons with musculoskeletal conditions

**DOI:** 10.1186/s12998-022-00466-w

**Published:** 2022-12-09

**Authors:** Joletta Belton, Hollie Birkinshaw, Tamar Pincus

**Affiliations:** Department of Psychology, University of Southampton, Southampton, UK

**Keywords:** Patient-centered, Reassurance, Validation, Communication

## Abstract

Consultations between practitioners and patients are more than a hypothesis-chasing exploration, especially when uncertainty about etiology and prognosis are high. In this article we describe a single individual's account of their lived experience of pain and long journey of consultations. This personal account includes challenges as well as opportunities, and ultimately led to self-awareness, clarity, and living well with pain. We follow each section of this narrative with a short description of the emerging scientific evidence informing on specific aspects of the consultation. Using this novel structure, we portray a framework for understanding consultations for persistent musculoskeletal pain from a position of patient-centered research to inform practice.

## Background

Effective patient-centred consultations are a collaboration between the healthcare professional and the patient, placing the person with pain at the heart of the interaction. This article is structured in a way that reflects this collaboration, showcasing the importance of acknowledging lived experience, the sharing of information, and the dynamic interactions that shape a consultation, in combination with supporting scientific evidence. The personal narrative in this article has been provided by Joletta Belton, who was a firefighter paramedic until persistent pain ended her career and upended the life she loved. We have structured the manuscript such that each section starts from Joletta’s personal narrative, followed with a concise review of supporting evidence. This method of co-production has the advantage of avoiding the biases and distortions that might result from researchers interpretation of others’ words. We have good reason to believe that the issues described by Joletta are common, as evidenced in the wealth of qualitative literature in this area [1, 2]. Our aim in using this format is to demonstrate the close link between personal narratives and emerging evidence, that combine to give an indication of better ways forward.

**Fig. 1:**
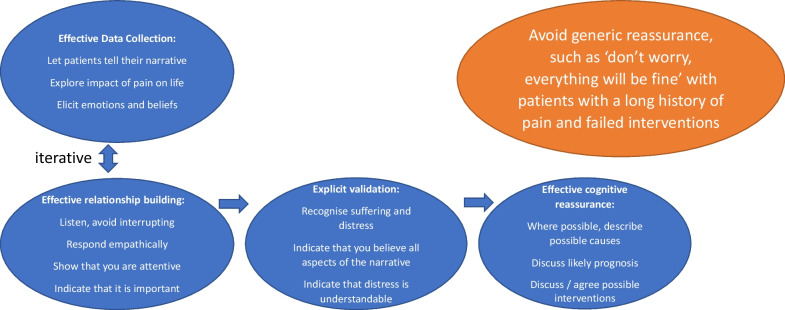
Effective and reassuring consultations

The sections below reflect the well-established model of reassurance originally proposed by Pincus and colleagues in 2013 [3], which suggests that effective and reassuring consultations include four components around good data collection, relationship building, avoiding generic reassurance and exchanging information, labelled as cognitive reassurance. The model has now been expanded to explicitly include a fifth component, the provision of validation, which emerged as a key component from qualitative work with patients [4, 5] (see Fig. 1). The methodology for the co-creation of this work was as follows: All authors familiarised themselves with the model of consultation-based reassurance. Each section of the narrative, following the components of reassurance described by the model, was then created by JB, followed by HB and TP reading it, and together writing up the evidence-base. The three authors then met to discuss the content and agreed amendments. This was carried out iteratively until all were satisfied.

### Pain and uncertainty: the role of effective reassurance

My pain began with an errant step and a twinge in my hip. Just a twinge. Yet that errant step set me on a path of ongoing, worsening pain that didn’t get better when it should have. Ongoing pain that didn’t make any sense. As a lifelong athlete who became a firefighter, I had experienced many injuries before this twinge, some for which I had to have surgery under general anaesthesia, and never once did I have a pain problem.

Why didn’t this pain respond to any of the treatments as expected, as it always had before? Why didn’t this pain respond to medications, physical therapy, injections, surgery, chiropractic care, acupuncture, massage, and more (I tried everything). I never received a satisfactory explanation, despite receiving many explanations that were often conflicting, both between different health professions and also within them, depending on the particular lens any given clinician was viewing my painful hip through. My pain was explained by dysfunctions (SI joint dysfunction, though I was told by other clinicians such a diagnosis did not exist, as well as various movement dysfunctions and, later in my journey, dysfunctional thoughts and behaviours). I was told my pain was due to damage, impingement, tears (a labral tear and annular tears), being ‘bone-on-bone’. Or that my pain was because I was out-of-alignment, my tibia was torqued, my spine was twisted, my hip (or core, spine, muscles) was unstable or imbalanced. With these many explanations there were many promised fixes, along with the high hopes that came with them, followed by devastating crashes when yet another promised fix didn’t work.

After years of so many failed treatments, treatments **I** failed in the vernacular of healthcare, I was lost, confused, uncertain. I had no idea what was happening in my body. The question of why I never recovered remained unanswered. No idea what to do about this pain that ended the career that had defined me. This pain that took me away from all of the people, places, and experiences that mattered to me. The ambiguity, the inability to make sense of things and thus an inability to find a way forward, was incredibly distressing. No one, and nothing, could reassure me. This all contributed a great deal to the painfulness of my pain.^1^

### The evidence: What is reassurance?

Although reassurance is mentioned in almost all guidelines for practitioners managing pain, evidence on reassurance was extremely scarce until 2008, when Linton and colleagues defined reassurance in a seminal paper in the journal Pain [6]. Linton et al., conceptualised reassurance as a set of behaviours, both verbal and non-verbal, that practitioners carried out with the aim of reducing concerns and anxiety in patients. Pincus and colleagues followed this with a body of research, including a review of current knowledge, the development of tools to measure and quantify reassurance [7], and subsequently, research that explored the association between reassurance and patient outcomes [8–10].

As there were insufficient studies at the time focusing on populations with pain, a systematic review was extended to include all consultations in primary care in which a strong level of self- management was indicated, and uncertainty about prognosis was likely to be high [10]. The review concluded that there was evidence for four distinct components of reassurance: Data Gathering; Relationship Building; Generic reassurance, and Cognitive Reassurance. Subsequent research has demonstrated that all of these are associated with patient outcomes. We discuss each component in turn.

The narrative above raises an important question: Is a full exploration of possible solutions and explanations necessary for patients to reach peace of mind, acceptance, adjustment and control? Or, in contrast, could such a costly journey be avoided, by a timely connection with a reassuring therapist? Although there is some evidence to suggest that people who rate their consultations as more reassuring seek less help later [3], there is a need to explore this further, in larger samples.

### Data gathering—hearing the whole story

#### Whose story? Whose goals?

When you look at my medical record, it tells the story of a painful hip, I am nowhere to be found. In fact, my medical record doesn’t seem to be about** me** at all. It is not** my** story. The only story I was allowed to tell in my care was very clinically-centred, made up of pain scales, marked up body charts, and questionnaires, rather than person-centred. Yet my pain, and the impacts of my pain, was so much more than that. In an attempt to get at the ‘important stuff’, what was important to me was largely glossed over, interrupted, or reinterpreted in ways that better suited the clinical story. My goals didn’t much matter either. Goals like being able to drive my car, go camping, go out to restaurants again, sit on the couch to watch a movie. Rather, clinically relevant goals were assigned to me, such as increasing my range of motion X degrees, doing X many sets of Y exercises, improving quad strength. There was a disconnect. Not a malicious or intentional one, but a disconnect nonetheless. One that could have been avoided if only my story had been heard and actively listened to, and if relevant questions that followed the story I told were asked. Then, a conversation could have taken place that would not only have helped the clinician to better understand me, my concerns, my personal contexts (and therefore better understand my pain and what we might do about it), it would have also have helped me to begin to better understand my pain and see possible ways forward.

### The evidence: effective listening

Early stages of the consultation typically involve data gathering and relationship building. Reassurance within these processes is largely implicit and non-verbal. Ideally, clinicians follow the narrative in the direction indicated by the person, ask relevant questions, and demonstrate they are listening and attentive through their eye contact, body posture, and demeanor. The flow of information at this stage is mostly from patient to clinician, yet clinicians need to indicate clearly that they are committed to hearing and understanding what is being said. Such commitment is implicitly reassuring, and aims to build trust, reduce anxiety, and create rapport [12]. Trust is needed because patients are more likely to heed advice given by a trusted source [13]; high levels of anxiety will impede patients’ ability to process information and make effective choices; and rapport is needed for patients to express their beliefs and concerns.

Despite this, patients often report that they feel the clinicians are not listening to them, or at least, not hearing their full story. Classic observational studies demonstrated that, on average, clinicians interrupted the patients’ narrative about their main concerns after around 18–23 s [14, 15]. Although more in-depth analysis indicated that not all interruptions are intrusive, and some aim at clarification, or rapport building [16], it remains concerning that physiotherapists have been shown to spend twice as long as patients talking in the first consultation [17], and that the most experienced practitioners were significantly more likely to talk over their patients [18].

### Relationship building—practitioners who care

#### Trusting, and being trusted

Trust goes both ways. We, the patient, need to trust the clinician who is treating us, and the clinician also needs to trust us, the patient. Through relationship building, communication, and conversation we move toward trust, respect, and a shared understanding of what is happening, which can then lead to shared decisions on the best treatment path forward. When we trust and are trusted, we know we are cared about, not just cared for.

Seven years into my pain experience I was asked by a practitioner, for the first time, to tell my story. I was taken aback, I didn’t know how to answer, so I asked where to begin. I was told to start wherever I wanted. I was surprised at the story I told. I gave voice to things I had never given voice to before, even to myself. And I was genuinely reassured that what I was experiencing, that what I was feeling—my fears, worries, concerns—were completely valid and reasonable. Of course I would feel that way! What I was going through was incredibly challenging and often scary! The difficulties of living with pain were acknowledged. My courage and strength and resilience were acknowledged. Being allowed to tell my story to a trusted and active listener who asked thoughtful and relevant questions, I gained some critical distance from what I was saying. I was able to start connecting some of my own dots, seeing new possibilities. We begin to make sense of what was happening, and to find possible ways forward, together. Throughout the encounter, what was most meaningful to me mattered. It was a profound and life changing encounter.

#### The evidence: empathy and the therapeutic relationship

An effective therapeutic relationship relies on establishing a meaningful connection between the practitioner and the patient [19, 20]. Indeed, for musculoskeletal pain, empathy is considered a fundamental component of good physical therapist interpersonal and communication skills; patients consider lack of empathy from the clinician as a major barrier to bonding [21]. There is a wealth of evidence that this empathetic therapeutic relationship is associated with improved clinical outcomes for musculoskeletal conditions (including pain and function), patient satisfaction, and adherence [22–25].

## Generic reassurance

### Being reassured, yet feeling abandoned

I was in a very vulnerable position when seeking care for my pain. Especially after seeing so many clinicians and trying so many things that did not work. It was on a roller coaster of emotions—the high hopes of promised fixes, and the devastating crashes when yet one more promised fix didn’t work. I thought I was to blame. And I felt such shame that I didn’t get better when I should have. I felt like I let everyone down. My family, my friends, my fellow firefighters. My healthcare professionals. My husband. Myself.

I was assured that everything that could be done was done, that there was nothing more that could be done, that the surgery was ‘successful’. This was far from reassuring. Reassurance in the absence of sense-making and validation was not helpful. ‘There’s nothing wrong’ was not reassuring when things definitely do not feel right, when my career had ended, when my self and my life were upended, when my future was lost.

#### The evidence: generic reassurance can be harmful

In contrast to common myths, the evidence suggests that non-specific empathic, reassuring statements expressed towards people living with chronic conditions are often associated with worse outcomes [3]. Subsequent research [4, 5] has clarified that though the empathic caring tone of these communications was very important to patients, the optimistic reassuring statements were perceived as patronising, and often turned out to be incorrect. Such communication, including statements such as ‘trust me, you will be fine’ and ‘I’ve seen this before, you have nothing to worry about’ result in immediate lowering of anxiety, but do not provide patients with new coping tools to manage their pain when it strikes again. This type of communication, referred to as Generic Reassurance, can foster dependence on serial consultations, and has been shown to be associated with worse outcomes, especially in those who are already experiencing distress [8].

## Validation

### Being believed—validating distress as normal. ‘You are not overreacting.’

Being believed, validated, acknowledged, heard and feeling seen and understood—that was life changing because a burden was lifted that I didn’t even know was there. There was immense relief in no longer having to ‘prove’ that I was in pain, that I was deserving of care, that I was a worthy human being.^2^ I was not fundamentally flawed. I was not to blame for my pain. I was not overreacting or too emotional. My pain was not ‘all in my head’. My pain was real. And once my pain was validated, once I, as a human being, was validated, it opened up capacity to take on new information. I could finally begin to think again, to plan, to remember, to act on what I’d learned. I could begin to incorporate that new information into my understanding of my pain, and myself with pain, and could see possibilities where before I saw only chaos, only losses, only pain. I could finally begin to tell a new story.

### The evidence: the impact of validation

Research has established validation as a critical component of an effective and collaborative therapeutic relationship between clinicians and people with pain. People with pain often report feeling that they are discredited, not believed, and that their pain is not legitimised by clinicians, especially if there is no underlying medical explanation, resulting in feeling unvalued as a person and contributing to a struggle in self-affirmation [28, 29]. When pain is validated by healthcare professionals, research shows that this is associated with greater patient satisfaction with consultations, improvements in mood, more open discussion and disclosure, and promotion of shared-decision making [2, 30, 31]. Promising evidence from experimental research even suggests that recall is improved after validation during a painful task [32]. Validation from healthcare professionals regarding the legitimacy of pain can provide the foundation to a collaborative relationship between clinician and patient who can then begin to move forward through the pain journey together, with trust and mutual assurance.

## Cognitive reassurance: mutual exchange of information towards the future

### Joint decision making

Importantly, my story was not merely replaced with a clinical story or a medical story or a pain science story, which had often happened in the past. That doesn’t work. In my experience, we cannot lecture people into a better understanding of their pain. We cannot just give them a new and better story. It has to be a conversation, an exchange of expertise to create, together, a narrative that makes sense of things in ways that are mutually acceptable. I am grateful that, with a trusted guide, I was able to co-create a new narrative that made sense of things in ways that made biological AND biographical sense.^3^ It was MY story, not a story of a painful hip. I was an active participant in the story’s creation. By bringing together my expertise, my lived knowledge of pain, together with what I have learned of pain science and my trusted guide’s expertise, knowledge, and skills, I was able to craft a narrative that not only made sense of things, it offered hope, and a way forward. Arthur Frank writes that we are the stories we tell ourselves, and ourselves are ‘being formed in what is told’[33].^4^ Our stories change as our understanding changes. This in itself can be therapeutic.

### The evidence: sharing decisions and facilitating moving forward

Toye et al. [2] developed a model of ‘moving forward alongside pain’ from a systematic review of 77 qualitative studies, which consists of strategies including ‘integrating my painful body’, ‘redefining normal’, ‘realising there is no cure’, and ‘becoming the expert’. Key to reaching acceptance and being able to move forward with pain is the understanding of pain itself. There is a robust body of evidence showing that cognitive reassurance—clinicians providing explanations and education about possible prognosis and management options—is one of the most effective methods for increasing sense of control, confidence, acceptance, trust, and patient satisfaction whilst reducing anxiety and feelings of isolation in people with pain [3, 36]. Explanations and education are likely to be most effective when integrated into conversations in the clinical encounter, rather than as general information about pain or pain science. The most relevant components of providing education, especially early on in pain experiences, may be the attention, interest, and active listening of the clinician [37]. Effective education and cognitive reassurance should be patient-centred; focusing on the patient’s story and concerns, and tailoring explanations to ensure understanding that aligns with the patient’s lived experiences. Together, these factors can then facilitate patients to move forward alongside their pain.

## Conclusion: how can consultations help patients embrace life?

Joletta: Over time, I came to understand my pain differently. More importantly, I came to understand MYSELF with pain differently. I could manage flare-ups without freaking out and thinking all progress had been lost. I could engage with the people, places, and experiences that mattered to me. I could live a joyful, meaningful, fulfilling life, even if pain was still present. Because pain was no longer the centre of everything. I was back at the centre of my story, of which pain is but a part.

This article has discussed a several recommendations for clinicians to use in consultations for chronic pain, which are summarised in Table 1. Ultimately, shared uncertainty between healthcare professionals and patients is the beginning of a journey together exploring the path of pain management. Accompanied by listening, validation, empathy and reassurance, a willingness to explore the paths together and accept the uncertainty of the journey is key to moving forward with chronic pain, for both healthcare professionals and people with pain.

**Table 1:** Recommendations for practice

Framework components	Application in practice
Data collection	Ask open-ended questions Allow patients to tell their story Check you understand what matters to them Check if you need to know anything else Try to avoid chasing hypotheses while people are talking Explore the whole person- don’t duck emotions, concerns and problems that are beyond your perceived scope
Relationship building	Make sure people know you have listened Show empathy for suffering, just as you would to a friend Become comfortable staying with patients’ distress
Avoiding generic reassurance	Avoid telling patients that everything will be alright unless you really know this is the case Recognize that telling patients nothing is wrong is not always reassuring
Validation	Be clear and explicit about the fact that you believe the patient Acknowledge the pain and the suffering Explicitly indicate that distress is completely normal under the circumstances
Cognitive reassurance	Discuss prognosis, treatment options, likely obstacles Use simple language and avoid jargon Make sure the conversation flows both ways Agree on ways forward

## Data Availability

We cite published data, but have collected no new data for this manuscript.

## Footnotes

1 JB comments: When I first read Drew Leder’s *The Experiential Paradoxes of Pain*[11]*,* I felt so seen and understood. He is able to capture many of the aspects of the experience of pain that make pain so painful, and so hard to explain.

2 In 2017 I wrote a blog post titled ‘Trying to get better while having to prove we’re in pain’[26]. A clinician who’d read the post shared with me a paper by Dr. Nortin Hadler titled ‘If You Have to Prove You Are Ill, You Can’t Get Well’ [27]. It was another instance of feeling seen and understood, yet also frustrated. It was written in 1996, over 20 years prior!

3 JB comments: I began framing my experiences in this way after reading some of John Launer’s work, particularly his book *Narrative-Based Practice in Health and Social Care: Conversations Inviting Change* [34]*,* as well as through the work of Howard Brody, particularly "My Story Is Broken; Can You Help Me Fix It?": Medical Ethics and the Joint Construction of Narrative [35].

4 JB comments: Arthur Frank’s book *The Wounded Storyteller* [33] helped me to better understand the power of narrative, especially the narratives we tell ourselves, when it comes to pain and illness.
